# Is mHealth a useful therapy for improving physical or emotional health in adolescents with cystic fibrosis? A systematic review

**DOI:** 10.1007/s12144-021-02452-6

**Published:** 2021-11-24

**Authors:** Selene Valero-Moreno, Laura Lacomba-Trejo, Inmaculada Montoya-Castilla, Marian Pérez-Marín

**Affiliations:** 1 Department of Developmental and Educational Psychology, Faculty of Psychology and Speech Therapy, Universitat de València, Av. Blasco Ibáñez, 21, 46010 Valencia, Spain; 2 Departament of Personality, Assessment and Psychological Treatment, Faculty of Psychology and Speech Therapy, Universitat de València, Av. Blasco Ibánez, 21, 46010 Valencia, Spain

**Keywords:** Adolescence, Cystic fibrosis, MHealth, Mobile applications, Systematic review, New technologies

## Abstract

Cystic fibrosis (CF) is a rare disease that severely compromises health and interferes with the lives of those who suffer from it and is especially challenging in adolescence. The use of tools such as MHealth may benefit the physical and psychological health of adolescents with CF. Therefore, this study aims to examine the benefits of MHealth in adolescents with CF through a systematic review. A search of the scientific literature following the PRISMA guidelines was conducted in the ProQuest Central, PubMed, Web Of Science, Embase and ínDICE databases, resulting in 186 studies, of which seven were selected (based on inclusion and exclusion criteria). Two blinded evaluators conducted the searches, the selection and data extraction process and the quality evaluation of the studies. The agreement between evaluators was excellent in all cases (Kappa ranged from .78 to .96). 214 pediatric CF patients (61.71% female) participated in the final analysis. The mean age was 12.76 years. The studies evaluated different types of mHealth tools, with greater homogeneity in the independent and dependent variables. The quality of the studies analyzed was poor, since these had small samples selected for convenience, conducted non-experimental and low-quality designs, recorded few variables, and their statistical analyses were not sufficiently robust. Further research is needed in this field, improving research designs and considering physical and psychological adjustment variables, as well as patients and family members in the process of health improvement.

## Introduction

Cystic fibrosis (CF) is a rare, genetic, chronic, multisystemic disease caused by a dysfunction of the CFTR gene (cystic fibrosis transmembrane conductance regulator), which alters ion transport in the apical membrane of epithelial cells in various organs and tissues. Its incidence varies from one to nine per 100,000 live births worldwide (Orphanet, 2019). It mainly affects the production of secretions from the exocrine glands, which become thickened and obstruct the drainage ducts, causing damage, infection, and inflammation. This disease can affect organs such as the lung, liver, pancreas and the reproductive system (Farrell et al., 2017). However, lung involvement and exocrine pancreatic insufficiency are the main factors responsible for the progression, severity, and mortality of patients. Thus, its medical treatment involves many specialists (CFS, 2019).

CF can have a major psychological impact on pediatric CF patients and their families. The fact of living with such this disease in adolescence, a stage that stands out for being of great changes in which the development of identity, the regulation of emotions, the quality of the relationship with the family and peers and sexuality/body development take place. It may have a greater impact on these patients. CF causes a general breakdown in the pattern of the adolescent’s daily life (change in routines, social roles, or limitation of recreational activities) (Cronly & Savage, 2019), the adolescent’s important self-concept of the body during this period may be affected, particularly because of growth deficits. The disease can influence social relationships, family, and friends, and cause them to feel different from their peers (Malone et al., 2019), and may be a threat to the need for independence among affected patients (Borschuk et al., 2016; Kaushansky et al., 2017). Their social life could be conditioned by the avoidance of smoke-filled environments. Also, avoid risky behaviors such as smoking or drinking alcohol. Behaviors that tend to appear in adolescence, and their avoidance may cause them to feel less accepted (Gérardin et al., 2018). Exacerbation of the disease can lead the adolescent to spend long periods of time away from their friends and educational environment, and feeling confined to the hospital or at home (Sunther et al., 2016). Their schedules are also altered by daily medical treatment, which requiresthem to perform aerosol exercises and physiotherapy both in the mornings and evenings (Helms et al., 2017). The psychological adjustment made upon disease progression and the challenging treatment regime that CF entails will become a relevant indicator of the patients’ health status, quality of life and future expectations (Cronly et al., 2019; Habib et al., 2015; Lerch & Thrane, 2019).

An appropriate approach to CF should be of a multidisciplinary nature. Along with medical and pharmacological treatments, psychological support will be essential (Sawicki et al., 2015). These patients will also need psycho-education and appropriate coping strategies for dealing with the difficulties they will encounter in their daily lives (dropping out or delaying school work, feelings of rejection, and physical, mental and emotional exhaustion due to everything involved in the disease process and in making decisions about their care) (Sawicki et al., 2015).

A hospital stay or visit is generally an unwanted event in anyone’s life, especially when the person is still a minor (Carrion-Plaza et al., 2020). This may hinder the leisure time that is an essential part of childhood-adolescent development or lead to a disruption of usual social interactions (especially in the case of long hospital stays). In addition, the adolescent may have to cope with boring and repetitive therapeutic procedures, or even undergo painful medical procedures (Eakin & Riekert, 2013), and all of these coupled with an increase in medical visits can sometimes pose a risk to their health because the risk of infections may increase.

Health interventions are currently being implemented through digital and technological platforms in patients with chronic diseases. The emergence of new technologies has provided a variety of tools that can be used as a means of creating patient-centred games and provide help during the current COVID-19 pandemic situation (Klein et al., 2020; Krasovsky et al., 2021). With the help of Information and Communications Technologies (ICTs), games and applications (Apps) can be developed to improve people’s physical and emotional health in a variety of ways (Carrion-Plaza et al., 2020): to increase motivation during therapy, provide distraction from a painful procedure, or provide a window for social development and interactions. In recent years, there has been growing interest in terms such as telehealth, telemedicine and mHealth. The term telehealth includes the full range of technologies and services to provide patient care and improve the healthcare delivery system (Poropatich et al., 2013). Telehealth differs from telemedicine in that it refers more broadly to remote healthcare services than telemedicine. While telemedicine refers specifically to remote clinical services, telehealth can refer to remote non-clinical services, such as continuing medical education and the management of meetings and providers (Poropatich et al., 2013). On the other hand, mHealth is, according to the World Health Organization (2011),“*the practice of medicine and public health supported by mobile devices such as phones, patient monitoring devices, digital assistants and other wireless devices*”. This includes lifestyle and wellness apps that connect people to medical devices or sensors, medication reminders and health information through telemedicine messages and services. This is where the present study will focus.

Study results show that these new technologies appear to have a positive impact on pediatric patients in terms of: a) improving: enjoyment, socialization, increased emotional expressions; and b) reducing: pain, anxiety, anguish and stress (Carrion-Plaza et al., 2020; Kruizinga et al., 2021; LeBlanc et al., 2013; Lu et al., 2018; Rudolf et al., 2019). However, there is still not enough evidence to thoroughly support these benefits, and more studies are needed to help scientifically validate the benefits of these therapeutic tools.

A study about patient apps to improve healthcare (IMS Institute for Healthcare Informatics, 2013) has performed one of the most comprehensive analyses on health apps to date. This study analysed 43,689 apps, showing that 45.8% were not really health-related, and most found no real benefits; 16.9% were reliable and aimed at professionals; and 37.3% were reliable and aimed at patients. There are therefore few Apps that provide guidance for the management of the disease, allow the recording of symptoms, or facilitate communication with professionals or expert patients. In the case of mHealth, there are studies that have analyzed its benefits in different chronic diseases. There are also studies that have done so in CF (Hilliard et al., 2014; Vilarinho et al., 2017), but they have focused on adults, not addressing the evolutionary stage of adolescence, hence the importance of this research.

For all of the above, there is still a major gap regarding specific developments aimed at paediatric patients, in terms of those whose main purpose is to cover therapeutic and not only evaluative objectives.

The aim of this study is, therefore, to determine whether physical or psychological benefits are observed in adolescents with CF after the use of mHealth tools. To achieve this, a systematic review focused on this objective will be carried out.

## Methods

This qualitative systematic review was conducted according to the Preferred Reporting Items for Systematic Reviews and Meta-Analyses (PRISMA) standard (Shamseer et al., 2015).

### Bibliography Search

The ProQuest Central, PubMed, Web Of Science, Embase and ínDICE databases were consulted by two authors (LLT, SVM) for relevant records published up to 22 February 2021. Based on the PICO approach (da Costa Santos et al., 2007), the following question was asked: Are there benefits compared to the control group (active or inactive) in psychological or medical aspects in adolescents (10–19 years old) diagnosed with cystic fibrosis using mHealth?

The final search combined the proposed key elements. The following Boolean expression was therefore used in ProQuest, Web of Science, PubMed and inDICES: ((mHealth OR telehealth) AND (adolescent*) AND (cystic fibrosis)) and in Embase, the following: ((‘mHealth’/exp. OR ‘telehealth’/exp. OR telehealth) AND adolescent* AND cystic AND fibrosis).

All the retrieved items were uploaded to Covidence (*Covidence systematic review software*, 2021), the online screening and data extraction tool. Duplicate articles were removed, after which two authors (LLT and SVM) reviewed the titles and abstracts of all the papers and excluded those articles that did not meet the inclusion criteria based on their title and abstract. The articles that were selected by either author, or which contained differences between their blinded decisions, were read in depth individually and blinded, and re-evaluated to judge their eligibility according to the inclusion and exclusion criteria. The same authors (LLT and SVM) inspected the reference lists of the selected studies to assess the inclusion of quality references that had not appeared in the initial searches. The manual search followed a snowball sampling procedure to identify relevant articles in the reference lists of potentially useful documentation. Finally, disagreements between the two authors were resolved by discussion. In this regard, a lot of thought was given to the fulfilment of all the inclusion criteria for each paper. A third researcher (MPM) was available to resolve ties if necessary. However, through discussion and reflection by the first two (LLT and SVM), differences were resolved.

**Fig. 1:**
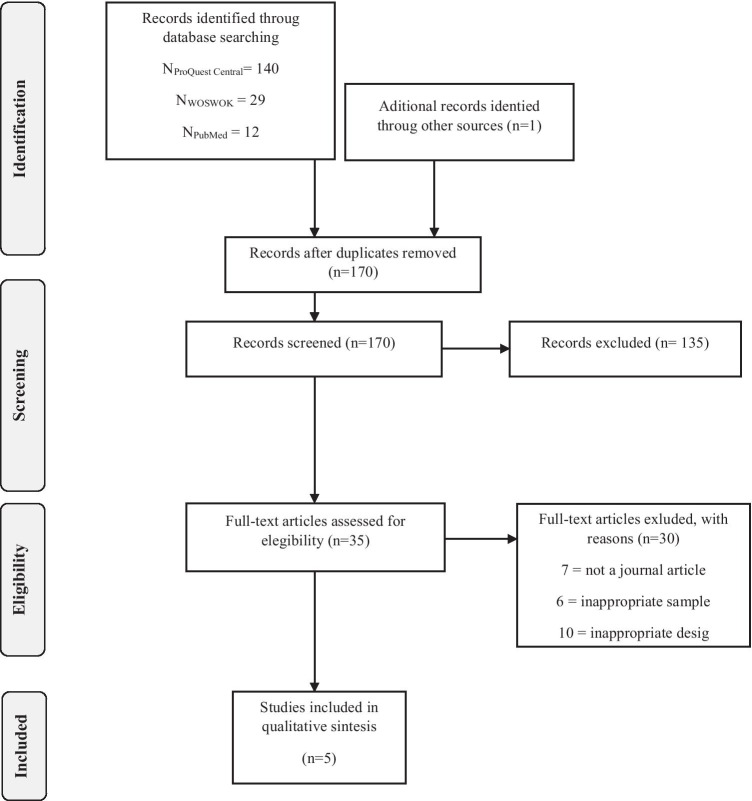
Flowchart of selection process

To evaluate the interrater agreement index, Cohen’s Kappa (κ) was used (Orwin, 1994), taking into account that values between −1 and 0.40 are considered unsatisfactory, values between 0.41 and 0.75 are considered acceptable and ≥ values of 0.76 are considered satisfactory (Hernández-Nieto, 2002). Figure 1 shows the flow chart of the information used to answer the review question. Due to the high degree of heterogeneity of these results, and specifically to the differences in the variables considered and the instruments used to evaluate them, a subsequent meta-analysis of these data was not considered appropriate, since they could not be combined.

### Inclusion and Exclusion Criteria

Studies that met the following criteria were included in the present systematic review: (a) the study assessed the impact of telehealth or mHealth on improving the physical or mental health of adolescents with cystic fibrosis. (b) the average age of the participants was between 10 and 19 years, (c) the study was published in journals with an impact factor, (d) the study was published in English or Spanish.

As exclusion criteria, it was suggested that the studies “could not” refer to: (a) people with other pathologies (b) papers published in conferences, (c) narrative reviews, (d) single case designs and (e) articles that if they covered several pathologies, did not break down in detail the results obtained for CF.

Finally, all the selected articles had to have appeared in the aforementioned databases (ProQuest Central, PubMed, Web Of Science, Embase and ínDICE), considering the above criteria, without applying any time limit.

### Quality Assessment

One author (LLT) developed a data extraction form that was used to obtain relevant information from the included studies. This information included the first author, journal of publication, year of publication, country, objectives, study design, sample size, mean age of participants, variables and instruments, type of intervention performed, results, main conclusions, and quality assessment rating (Tables 1 and 2).

**Table 1:** Characteristics of studies included in the systematic review

First author	Year	Country	Objectives	Sample	Variables and instruments	Design	Results	Conclusions
Bella	2009	Italy	-Assess the effect of telehomecare in CF patients, by systematically monitory respiratory parameters.	45 adolescents with CF 10–19 years (14.77 ± 5.22)	-Sociodemographic (sex, gender). -FEV1 value over time. -Outpatient visit rate and hospitalization rate -Rates of intravenous antibiotic therapy -Compliance: number of days in which patients transmitted overnight SaO2 value.	Descriptive non-experimental, longitudinal	-The average compliance (transmissions/patient/day) was 52.4%. -THC patients had a higher outpatient visit ratep (p = 0.024) and a higher number of therapy cycles (p = 0.01) compared with controls during the follow-up. -Both groups showed a progressive decrease of FEV1 over time and no statistically significant difference was detected when comparing the two groups over time.	-Receiving many concurrent therapies may affect compliance rates. -Patients treated with telehealth can obtain greater stability in lung function -Lack of patient acceptance of these types of healthcare tools.
Chrysochoou	2017	Greece	-To evaluate the safety and effectiveness of a home care programme for children with CF and to assess the value of regular telephone contact with the CF	60 children with CF (13.25 ± 2.62)	-Sociodemographic (sex, gender, distance to hospital). Exacerbations, respiratory infections or symptoms, weight gain, medication, and treatment adherence. -Expiratory volume in the first second (FEV1)	Descriptive non-experimental, longitudinal	-The QoL parameters and the total QoLscore improved significantly after the programme started, with a mean (SD) baseline QOL score of 39.88 (8.48) and mean (SD)delta QOL score of 43.44 (14.50) (p < .001) -FEV1%predicted showed a significant improvement -The days of hospitalization decreased significantly, from a mean (SD)baseline of 5.40 (6.06) days to a mean delta(SD) of 1.65 (2.14) (p = .02) -The costs also decreased significantly from a mean baseline (SD) of €2053 (1429) to a mean delta(SD) of 431.00 (565.4) (p = .005) -No significant differences were found between the two groups in FEV1and the days and cost of hospitalization after the implementation of the home care program	-Regular use of the telephone or these types of tools is effective especially for patients who live further away from the hospital. -Regularly collecting medical information using telephone calls allows clinical support teams to comprehensively monitor patients with chronic conditions and adjust their treatment. - The home care program and telephone communication significantly improved the QoL, treatment adherence and lung function of children with CF.
Modi	2006	U.S.	- To document rates of adherence to medical regimens for children with CF using four methods of measurement. - To assess convergence across the different adherence measurement methods and identify the measure that correlated most strongly with electronic monitoring.	37 children with CF 6–13 years (10.10 ± 2.5) and their principal caregivers	- Sociodemographic (age, gender, socioeconomic status, occupation, and composition of the family). Clinical (patient and caregiver medical history, presence of diseases in other children). - Activity record (type, duration, and company) in the last 24 h of the parents, in addition to the state of mind at that time through the prescribed treatment plan - Adherence: (1) The prescribed treatment plan (PTP (Quittner et al., 2000); 2) The disease management Interview-CF (DMI-CF) (Rand, 2000), (3) Refill data from the patient’s pharmacy. (4) Daily phone diary. (5) Electronic monitoring/counter method (only enzyme medications). - Health status (lung function tests).	Descriptive non-experimental, longitudinal	- Therapeutic adherence rates vary depending on the treatment component and the measure. - Adherence in children and adolescents with CF assessed with objective measures is low (<50%) compared to self-reported measures of children and parents (80%). - Adherence rates tend to be higher when self-reported and lower when monitored electronically. - Statistically significant (p < 0.05) difference in parental communication of adherence to enzyme medication compared to pharmacy refill history, diary data and electronic monitoring.	- High variability among methods of adherence evaluation. - Self-report measures indicate greater adherence than objective measures. - Low therapeutic adherence, which may put the health of adolescents with CF at risk. - Results from objective measures are comparable to each other, but not to self-reports. - Parental self-reports are consistent with those of their children. - Need to examine adherence by treatment component.
Marciel	2010	U.S.	- To assess through the perspective of the CF interdisciplinary care team, patients and parents, the acceptability, feasibility and usefulness of a mobile application (CFFONETM) for adolescents with CF. - Determine the optimal content and format for CFFONET. - Collect expert opinions on the technical design and feasibility of CFFONETM - Build and evaluate the usefulness of a CFFONETM prototype.	17 health professionals (n = 17), 12 adolescents with CF aged 11–18 (M = 15.7; SD = 2.23) of which 9 tested the prototype (M = 13.9; SD = 2.23). 6 adults with CF aged 21–36 (M = 30.70; SD = 4.72), 12 parents of adolescents with CF age from 41 to 54 years (M = 45.80; SD = 3.36) and 8 technology experts.	- Sociodemographic (age, sex). - A focus group with health care professionals from the pediatric and adult CF care teams. - Structured interviews and rating scales administered to adolescents with CF, adults with CF, parents of adolescents with CF and technology experts, all elaborated ad hoc, four your opinion about the App.	Descriptive non-experimental, cross-sectional, retrospective assessment of adults with CF	- Adolescents have less therapeutic adherence and knowledge of the disease than other age groups. -Both health professionals and technology experts rated the prototype positively. - Healthcare professionals felt that the platform was moderately effective in increasing knowledge, social support and to a lesser extent improving treatment adherence. - Adolescents showed great impairment from CF in their daily lives and reported barriers to adherence, with parents reporting greater difficulties. - Adults with CF showed similar concerns to parents of adolescents with CF. CF patients (adolescents and adults) perceived CFFONETM as helpful. Chatbot (connecting with other adolescents with CF) was highlighted as potentially useful. - Technology experts assess CFFONETM as very feasible and appropriate for adolescents.	- The tool can be useful to improve therapeutic adherence, considering the experiences of: health personnel, patients and caregivers. - The platform could include recommendations to improve family functioning.
Morton	2017	U.K.	Investigate whether regular text message reminders sent from existing hospital automated systems can improve adherence to nebulized therapy in children with CF	13 pediatric patients with CF, aged from 6 to 15 years (M = 12).	- Sociodemographic (sex, age). - Clinical: nebulized medications, adherence (percent of prescribed doses taken). - Participants’ opinions on receiving text message reminders and their perceived utility (questionnaire elaborated ad hoc).	Descriptive non-experimental, longitudinal	- Adherence to nebulizer therapy is high (80–81%). - There was no difference between time 1 and time 2 (patients were their control group using the waiting list format). - Messages did not change the rates of adherence to nebulized medication (p > .05 in all comparisons). - Adherence in patients with high (baseline) adherence was maintained, in those with moderate adherence it increased slightly, and in those with poor adherence it worsened.	- Sending reminder messages for medication is feasible. - Adherence may be improved in patients with moderate adherence.
Rudolf	2019	Germany	- Assess the behavior and satisfaction of adolescents and youth with CF from an mHealth application. - Identify areas of improvement for CF. -Compare general and disease-specific satisfaction, lung function, and anthropometry before and after using the application.	25 adolescents and young adults with CF (age range 12–24) (M = 16.00; SD = 3.00)	- Sociodemographic (age, sex) - Clinical: Lung function tests and body mass index. - Psychological: life satisfaction (FLZ; questionnaire on life satisfaction (Goldbeck et al., 2003). - The use of the applications (System Usage Scale) - The quality of the product (AttrakDiff questionnaire) (both created ad hoc) - Product’s usability and design (Hassenzahl, 2009)	Descriptive non-experimental, longitudinal	- The use of the mobile application (MP) was not very frequent. - What they used most (and found useful) was medication reminders. - Most felt that the PPP helped them in their health management but also personally. - However, it did not improve their satisfaction with life or health (p < .05). - The use of PPP improves or stabilizes the subitem respiration and the subitem lack of handicap by CF (p < .05). - Lung function and anthropometry were not modified by its use (p > .05)	- Most of the patients did not want to continue using the app after the study period. - Only a few CF-specific aspects of weighted life satisfaction were stabilized by the mHealth app; clinical parameters were not affected. - Adaptation of the functions to adolescent-specific needs could improve the long-term use and thus positively affect the disease course.
Logie	2020	Australy	- Assess the feasibility of spirometry assessments using telehealth. - Assess if telehealth spirometry could support the CF team during ‘home admissions’ and for ongoing home monitoring in children living outside.	25 pediatric patients with CF (7–17 years) (M = 10)	- Sociodemographic (sex, age). - Clinical: lung function tests, child’s medical record.	Descriptive non-experimental, longitudinal	-Telehealth spirometry was successful most of the time (93%). - It resulted in very large savings in time and finances. - In addition, it allowed the detection of critically ill patients who were immediately hospitalized.	- Telehealth allowed these impairments to be detected earlier than they would otherwise have been under standard care and led to accelerated hospital admissions. - It also facilitated earlier than expected discharge of patients who recovered.

**Table 2:** Article quality assessment

First author	Study design	Blinding	Representativeness selection bias	Representativeness withdrawals and dropouts	Confounders	Data collection methods	Data analysis	Reporting	Total	Overall quality assessment
Bella	3	3	3	3	1	3	3	3	2.75	Weak
Chrysochoou	3	3	3	3	1	2	3	2	2.50	Weak
Modi	3	3	3	1	1	1	2	2	2	Weak
Marciel	2	3	3	1	1	3	3	2	2.25	Weak
Morton	2	3	3	1	1	1	1	2	1.75	Weak
Logie	3	3	3	1	1	2	1	1	2	Weak
Rudolf	2	3	3	1	1	2	2	2	2.75	Moderate

### Data Extraction

Two authors (LLT and SVM) independently and blindly assessed the quality of the included studies using an adapted version of the Quality Assessment Tool for Quantitative Studies developed by the Effective Public Health Practice Project (Wermelinger Ávila et al., 2017). This tool consists of 19 items that assess 8 criteria: (a) study design, (b) blinding, (c) representativeness – selection bias, (d) representativeness – withdrawals and dropouts, (e) confounders, (f) data collection methods, (g) data analysis, and (h) reporting. The rating for each criterion ranges from 1 (low risk of bias; strong) to 3 (high risk of bias; weak). Based on the study by McMullan (McMullan et al., 2019), studies can have between 4 and 8 component ratings based on the 8 criteria. An overall rating is assessed according to the component ratings. For example, a study with 6 ratings could be rated as “strong” if there are no WEAK ratings and at least 3 STRONG ratings, “moderate” if there is one WEAK rating and less than 3 STRONG ratings, or “weak” if there are two or more WEAK ratings.

## Results

### Study Selection and Screening

The study selection process is shown in Fig. 1. The literature search resulted in a total of 206 records. After removing duplicates, the total number of records was 189. The initial selection excluded 189 studies based on title and abstract, and the full content of the remaining 42 papers was read in a second selection process. The reliability of the prior agreement between the two independent reviewers (LLT and SVM) in the full-text screening was excellent (κ = 0,78). In the second screening, 30 papers were excluded and thus 7 dependent studies were eligible for inclusion. The degree of inter-judge agreement was also excellent in this second screening (κ = 0,88).

### Characteristics of the Study

The study characteristics are summarized in Table 1. The seven studies investigated included a total of 214 pediatric CF patients, of whom 61.71% were female. One study did not report the percentage of people of each sex. The number of participants in the studies ranged from 12 to 60, with an average sample size of 29.14 participants. The average age of participants ranged from 6 to 24 years with an average of 13.26 years. Forty percent of the studies included the primary caregivers of adolescents with CF. Of these studies, one study also included adults with CF who commented on their experience as teenagers, as well as CF health professionals and technology experts.

In terms of the research design, no study was randomized, nor were participants or investigators blinded. Six were longitudinal studies, and five of them had conducted two evaluations (one before and one after the intervention). The average evaluation time in these studies was 8 months, with a range of 2 to 24 months between evaluations. One study was cross-sectional, with an evaluation at a single point in time.

Regarding the representativeness of the sample for treatment dropout, two of the studies did not report the dropout or withdrawal rate among the participants. In the other studies, the average dropout rate was 27.17% in the pre-intervention period (range: 0% to 43,30%) and 0% in the post-intervention period, since in the studies in which individuals withdrew, they did so before the intervention because they were unwilling to participate in several evaluations over time.

There was considerable heterogeneity in terms of the independent and dependent variables assessed. The studies considered various physical and psychological adjustment outcome variables, including the parental activity record (type, duration and company) in the last 24 h, as well as their current state of mind in relation to the prescribed treatment plan (Rand, 2000), their opinion on the usability and feasibility of the App (one study), their opinion on the SMS reminders for the nebulizer therapy (one study), adherence, outpatient visit rate and hospitalization rate, rates of intravenous antibiotic therapy and exacerbations.

Two of these studies evaluated the opinion of CF patients, and that of other people who are also relevant to the treatment (such as family members, health professionals and technology experts), one of them used a tool created an ad hoc. Adherence was assessed using objective methods (five studies) (patient pharmacy refill data, electronic monitoring method only considering enzyme medications, the percentage of prescribed doses of nebulized medications taken, lung function test, body mass index and FEV1) and subjective criteria (compliance with the prescribed treatment plan (Quittner et al., 2000), the disease management interview (Rand, 2000) and daily phone diary). The most widely used method of assessing adherence was the lung function measurement by spirometry (five studies).

However, only one study evaluated psychological aspects of adaptation, considering life and health satisfaction as psychological variables (Rudolf et al., 2019). The assessment was a self-reported measure, and the psychometric properties of this instrument are not reported in the work.

One study used mHealth in comparison with other measures of adherence (self-reported) among the same patients. Twenty percent of the control patients were selected after the evaluation of adolescents in the experimental group. One study compared mHealth with usual care in different patient groups. In two studies, the control group consisted of the patients themselves with a waiting list format, and two studies did not use a control group. Two evaluators (LLT and SVM) assessed the presence or absence of a control group and the presentation format, the inter-rater agreement reliability reached an almost perfect level of agreement (*κ* =,96).

Only one study consulted the medical history of the adolescents and their caregivers to control for confounding/contaminating variables (e.g. the presence of pancreatic insufficiency, pseudomonas and its frequency (chronic or intermittent). However, the data obtained from the consultation conducted were not reported. Regarding the therapy format, all studies used mHealth individually, although one of them had the option of a chatbot with which participants could communicate.

**Fig. 2:**
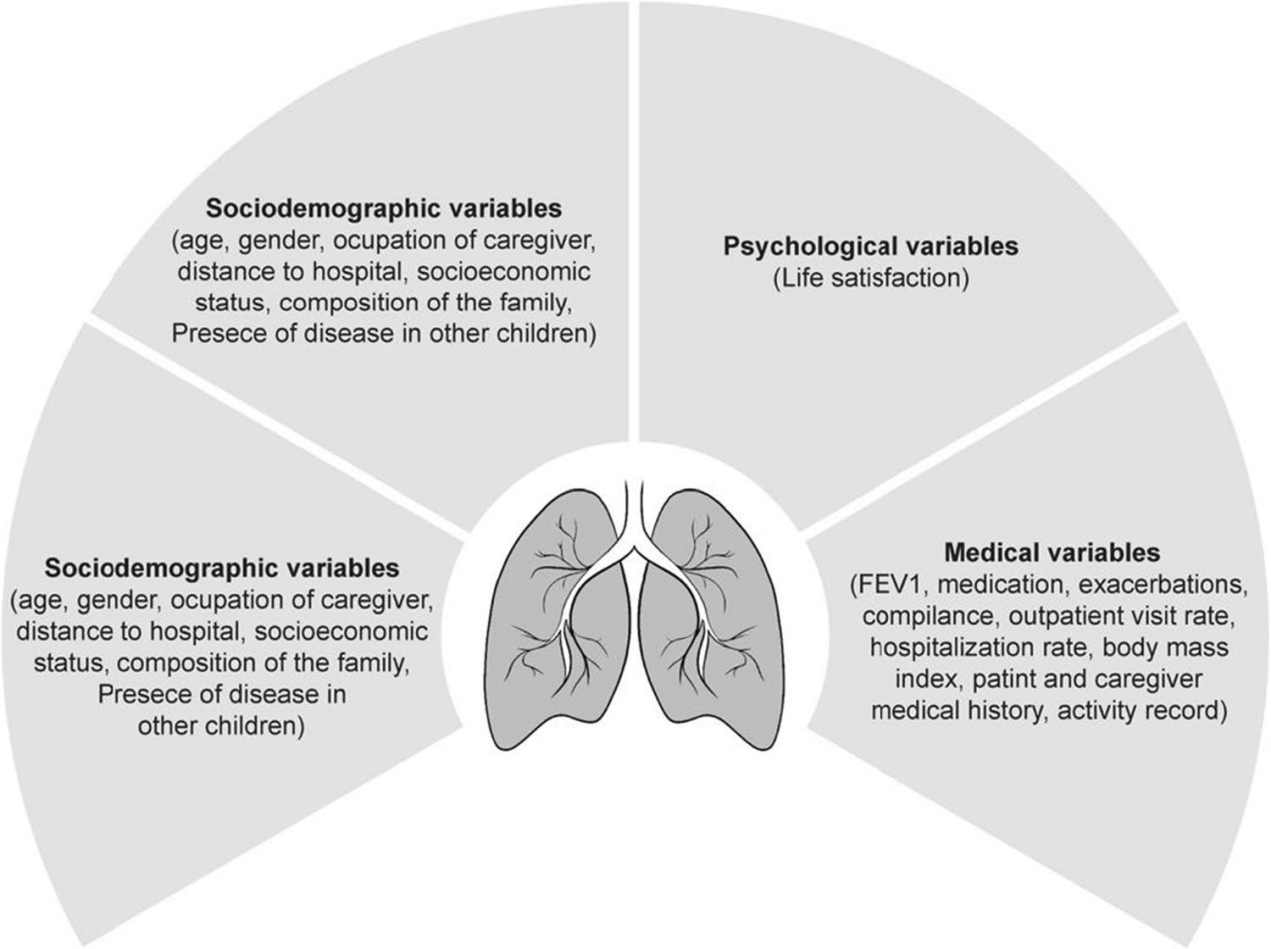
Summary of the main results

All the studies accessed the sample by means of convenience sampling and evaluated the participants when they attended their medical appointments. A total of 42.85% of the studies showed their inclusion criteria. In this regard, one study mentioned the need for adolescents and their caregivers to have a good knowledge of English, one study focused on the age of the participants (12–24 years), one study requested that patients had previous poor control (multiple infectious pulmonary relapses) and a significant decrease in FEV1 values (more than 10%) and another one focused on the need to have been using spirometry for 7 years or longer and to have access to an electronic device to perform it. The remaining studies did not clearly specify the inclusion and exclusion criteria for the participants. A summary of the results can be found in Fig. 2.

### Quality Assessment

Table 2 shows the quality ratings of the study. The average quality score was 2.59, with quality scores ranging from 1 to 3, with 1 being the highest score (least likely to be biased and highest quality) and 3 being the weakest score (most likely to be biased or lowest quality). The assessment of study quality was conducted independently by two evaluators (LLT and SVM) and resulted in a strong level of agreement (κ = 0,95).

## Discussion

This study aimed to determine whether physical or psychological benefits are observed in adolescents with CF after they use mHealth tools by means of a systematic review. After an exhaustive search, a total of seven studies were included for the analysis of the use of mHealth in CF patients. These studies focused above all on the physical benefits, but did not analyse their impact on psychological aspects, showing the need for continued work in this area due to the scarcity of research in this field. The variables assessed in the studies included: socio-demographic variables (mainly age and gender), medical variables (FEV1, medication, exacerbations, compliance, outpatient visit rate, hospitalization rate, body mass index, patient and caregiver medical history, activity record), other variables (mainly experience and opinion about the treatment) and one study took into account a psychological variable (life satisfaction). For more information, see Fig. 2.

The applications presented are diverse, ranging from the monitored recording of some biological markers (such as enzyme medication) to reminders of the need for daily application of nebulized therapy via text message. Other studies (Marciel et al., 2010; Morton et al., 2017) consider the opinions of the participants, as well as other experts in the field (patients, family members, healthcare personnel specialized in the care of people with CF and computer experts).

Three studies (Bella et al., 2009; Chrysochoou et al., 2017; Logie et al., 2020), describe the usefulness of teaching patients to perform spirometry at home, highlighting the medical and economic benefits (in terms of the time saved and the economic resources invested in attending the medical center). In addition, it mentions the benefits involved in the early detection of any problems in routine control and possible earlier discharge. However, the results are also inconclusive.

Finally, a study (Modi et al., 2006) reports data of great interest regarding the application of mHealth, since it examines the differences between subjective measures (or self-reported and reported by caregivers) and objective measures based mainly on mHealth, considering that objective physical adaptation is much lower than subjectively reported adaptation, and as such the use of both methods is recommended. The objective measures in the study are also considered to be comparable to each other.

The results of these studies are not conclusive, as they all use small and heterogeneous samples. None of them provide extensive detail on variables that could confound/contaminate the results, such as the presence of pancreatic insufficiency, pseudomonas and its frequency (chronic or intermittent). Furthermore, as noted in the evaluation of the quality of the studies, all the studies are descriptive, and only two investigations conducted a t-test comparing the pre- and post-treatment measures, or the experimental group with the control group. Only one study (Rudolf et al., 2019) adds the calculation of the effect size, which is a very important statistic in this type of studies, considering the sample sizes involved. No study uses robust research and statistical designs that would allow the results obtained to be analysed consistently.

The results of these studies may have been affected by some biases, including the following: biases in the research design (lack of randomization in the participants, no double-blind assignment between the control group and the experimental group; no control group in some cases, a lack of a robust statistical methodology to adequately analyze the benefits of the interventions, and most of the studies are cross-sectional or with short follow-up times after the intervention). In addition to the above, there could be biases in the variables or instruments used, as few studies assess both physical and psychological adjustment. Furthermore, as already mentioned, the medical evolution of these patients is inadequately recorded,and there is a lack of control of the variables used in the studies. On the other hand, no study included any other intervention technique that would allow comparison of mHealth with other techniques. If different interventions had been included, it would have been possible to assess whether losses in the sample were due to the method used or to other causes. Finally, there could be biases in the selection of participants, since tools applied to the specific stage of the life cycle have not been used; in most studies, children have been mixed with adolescents and adolescents with adults, with no distinction made for the use of these tools according to the specific needs of the sample.

Despite the possible biases, it must be acknowledged that conducting a study of these characteristics is very difficult, as well as accessing hospital-type samples, and paediatric patients with a rare disease such as CF is particularly complicated. Despite the small sample size associated with the prevalence of this disease, there is some homogeneity in the number of participants among the studies included in the review. Another positive aspect is that in the studies where experimental mortality is reported, the rate of treatment dropout is low, which is beneficial because it could indicate that the use of these tools could motivate adherence in adolescence.

We therefore believe that these types of tools are an opportunity in terms of time savings and direct economic cost reduction for this group of patients and their families (Morton et al., 2017), and provide the possibility of medical tests such as home spirometry. Moreover, the use of mHealth currently allows access to the control and evolution of their health through Apps, as well as communication with healthcare personnel, without the need to visit health centers and hospitals.

Despite the various strengths of this systematic review, several limitations should be taken into consideration when interpreting its results. The studies included were mainly based on non-randomly selected samples and with an absence of variable control. These studies are prone to sampling bias. The heterogeneity of the measures used makes it impossible to perform a meta-analysis of the data, although it would be desirable to do so in the future. Furthermore, the strict inclusion criteria may have resulted in the exclusion of some relevant articles. However, this strategy enhanced the possibility of comparisons between the included studies. The cross-sectional design of most studies did not allow conclusions to be drawn about the true benefits of the mHealth intervention in adolescents. Analyses based on longitudinal studies are therefore the most desirable approach for future research, providing further clarification and evidence of the quality of the relationship between physical and psychological health variables in adolescents with CF.

To our knowledge, this is the first systematic review conducted on the possible benefits of mHealth in adolescents with CF. As a result of systematic research of the literature based on precise inclusion criteria and systematics, this review broadens the knowledge beyond the conclusions of the narrative reviews. Additionally, this review included two blinded evaluators throughout the process, as well as the agreement index between them. Based on the results obtained, it is necessary to conduct research that assesses psychological and medical aspects, considering an integrative and multidisciplinary approach to health from a biopsychosocial point of view. Future research should include the assessment of psychological aspects such as protective factors (self-esteem, emotional competences, family styles, resilience or social support), risk factors (perceived stress, presence of psychopathology), adjustment variables to the disease such as health-related quality of life, perceived level of threat or coping strategies. There is also a need for this type of intervention to be directed not only at the patients, but also at their family system.

We therefore conclude that more research on CF apps in the hospital context is required, since it is an area of great interest that has been the focus of few studies and is showing satisfactory results. However, the quality of the research designs used should be increased to adequately assess their effectiveness.

## Data Availability

The datasets generated during and/or analysed during the current study are available from the corresponding author on reasonable request.
